# FAIM Enhances the Efficacy of Mesenchymal Stem Cell Transplantation by Inhibiting JNK-Induced c-FLIP Ubiquitination and Degradation

**DOI:** 10.1155/2022/3705637

**Published:** 2022-09-30

**Authors:** Jinyong Chen, Feng Liu, Wangxing Hu, Yi Qian, Dilin Xu, Chenyang Gao, Zhiru Zeng, Si Cheng, Lan Xie, Kaixiang Yu, Gangjie Zhu, Xianbao Liu

**Affiliations:** ^1^Department of Cardiology, The Second Affiliated Hospital, Zhejiang University School of Medicine, Hangzhou 310009, China; ^2^Cardiovascular Key Laboratory of Zhejiang Province, Hangzhou 310009, China; ^3^Department of General Intensive Care Unit, The Second Affiliated Hospital, Zhejiang University School of Medicine, Hangzhou 310009, China; ^4^Department of Rheumatology, The Second Affiliated Hospital, Zhejiang University School of Medicine, Hangzhou 310009, China

## Abstract

**Background:**

The poor survival rates of transplanted mesenchymal stem cells (MSCs) in harsh microenvironments impair the efficacy of MSCs transplantation in myocardial infarction (MI). Extrinsic apoptosis pathways play an important role in the apoptosis of transplanted MSCs, and Fas apoptosis inhibitory molecule (FAIM) is involved in regulation of the extrinsic apoptosis pathway. Thus, we aimed to explore whether FAIM augmentation protects MSCs against stress-induced apoptosis and thereby improves the therapeutic efficacy of MSCs.

**Methods:**

We ligated the left anterior descending coronary artery (LAD) in the mouse heart to generate an MI model and then injected FAIM-overexpressing MSCs (MSCs_FAIM_) into the peri-infarction area in vivo. Moreover, FAIM-overexpressing MSCs were challenged with oxygen, serum, and glucose deprivation (OGD) in vitro, which mimicked the harsh microenvironment that occurs in cardiac infarction.

**Results:**

FAIM was markedly downregulated under OGD conditions, and FAIM overexpression protected MSCs against OGD-induced apoptosis. MSCs_FAIM_ transplantation improved cell retention, strengthened angiogenesis, and ameliorated heart function. The antiapoptotic effect of FAIM was mediated by cellular-FLICE inhibitory protein (c-FLIP), and FAIM augmentation improved the protein expression of c-FLIP by reducing ubiquitin–proteasome-dependent c-FLIP degradation. Furthermore, FAIM inhibited the activation of JNK, and treatment with the JNK inhibitor SP600125 abrogated the reduction in c-FLIP protein expression caused by FAIM silencing.

**Conclusions:**

Overall, these results indicated that FAIM curbed the JNK-mediated, ubiquitination–proteasome-dependent degradation of c-FLIP, thereby improving the survival of transplanted MSCs and enhancing their efficacy in MI. This study may provide a novel approach to strengthen the therapeutic effect of MSC-based therapy.

## 1. Introduction

Mesenchymal stem cells (MSCs) transplantation has been shown to be safe and beneficial in the treatment of myocardial infarction (MI) due to the low immunogenicity, multidirectional differentiation ability, and robust paracrine effects of these cells [1, 2]. Several studies have revealed that the main factor that impairs the therapeutic efficacy of MSCs transplantation is the poor survival rate of transplanted cells [3], which is caused by cell death induced by a harsh microenvironment and the limited self-renewal rates of MSCs [4]. A previous study reported that Fas-FasL interactions have a significant effect on death receptor (DR) activation in implanted MSCs, and that inhibiting this interaction by recombinant Fas/Fc protein treatment improves cell retention and restores cardiac function [5], suggesting that extrinsic apoptosis pathways play an important role in transplanted MSCs apoptosis and that intervening in these pathways by genetic modification may be a new strategy to augment the longevity of transplanted MSCs.

Originally identified as an inhibitor of Fas signaling in B-cell receptor-activated lymphocytes [6], Fas apoptosis inhibitory molecule (FAIM) plays an essential role in apoptosis, metabolism, cell growth, and tumorigenesis [7]. Studies have revealed two splice variants of FAIM, and the short variant is ubiquitously expressed in multiple cell types [8–10]. Increasing evidence indicates that FAIM exerts an antiapoptotic effect on thymocytes and hepatocytes by suppressing T-cell receptor (TCR) signaling or DR signaling [11, 12]. FAIM has been implicated in the regulation of heat- and oxidative stress-induced cell death, and FAIM knockout cells exhibit impaired resistance to cell death induced by stress [13]. A recent study reported that FAIM facilitates cell proliferation by suppressing autophagy [14]. We therefore hypothesized that FAIM might be an excellent candidate to promote MSCs survival and optimize the abilities of MSCs in a hostile ischemic environment.

Cellular FLICE inhibitory protein, also known as c-FLIP, is a strong regulator of the extrinsic apoptosis pathway and competitively inhibits the binding of caspase-8 with FADD, thus avoiding the activation of caspase-8 [15, 16]. c-FLIP expression is markedly decreased in thymocytes and hepatocytes in the absence of FAIM [17]. To date, however, the underlying mechanisms linking FAIM to c-FLIP expression remain unclear, necessitating further exploration.

In this study, we discovered that FAIM overexpression promoted MSCs survival and enhanced their therapeutic effect in MI. The potential mechanisms involve reducing the ubiquitination and degradation of c-FLIP mediated by impairing JNK activation. Our results provide new insights and alternative treatment strategies for stem cell transplantation.

## 2. Materials and Methods

### 2.1. Cell Culture and Characterization

Mouse bone marrow-derived MSCs were purchased from Procell (Wuhan, China). Cells were cultured in low-glucose DMEM (#C11885500BT, Gibco, CA, USA) supplemented with 10% (v/v) fetal bovine serum (FBS) (#10270-106, Gibco) and 100 U/ml penicillin/streptomycin (PS) (#15140-122, Gibco) in a 5% CO_2_ humidified atmosphere at 37°C.

MSCs were characterized by flow cytometry to analyze the expression of membrane markers with a mouse MSCs surface marker assay kit (#MUXMX-09011, Cyagen Biosciences, Suzhou, China). In brief, MSCs were digested with trypsin (Genom Bio., Hangzhou, China) and resuspended in Hank's balanced salt solution (HBSS) (Genom Bio.). Subsequently, the single-cell suspensions were incubated with cell surface marker antibodies at 4°C for 30 min (mesenchymal surface markers: Scr-1, CD29, CD44, and CD90; hematopoietic stem cell marker: CD117; endothelial cell marker: CD31; and isotype-matched control). After being washed with HBSS, the MSCs were incubated with fluorescence-conjugated secondary antibodies for another 30 min. Surface marker expression was analyzed with an LSRFortessa flow cytometer (BD, USA). The MSCs were positive for CD29, CD44, CD90, and Sca-1 and negative for CD31 and CD117, and the MSCs were fusiform and arranged in a swirled pattern (Supplementary Figure S1(a)-1(b)).

### 2.2. Lentivirus Infection

Recombinant lentiviruses expressing mouse FAIM-S labeled with GFP and Flag (GFP-FAIM) or mouse c-FLIP labeled with GFP (GFP-FLIP) and empty lentivirus labeled with GFP (GFP-Vec) were provided by GeneChem (Shanghai, China). MSCs were transduced with lentiviruses (MOI = 50) overnight with HitransG A (GeneChem, Shanghai, China) after being seeded in flasks. Then, the virus-containing supernatants were replaced with fresh medium for further culture. The infection efficiency was determined by measuring the expression of the target gene and the fluorescence of GFP.

### 2.3. siRNA Transfection

Small interfering RNA (siRNA) oligonucleotides targeting mouse FAIM (si-FAIM) or mouse c-FLIP (si-FLIP) and scramble siRNA (si-Scr) were synthesized (Tsingke, Hangzhou, China) and transfected into MSCs with RNAiMAX reagent (#13778-150, Invitrogen, CA, USA). Twenty-four hours after transfection, the transfection efficiency was measured by Western blotting. The siRNA sequences are listed in Table 1.

### 2.4. Cell Apoptosis Model

To simulate the microenvironment of the ischemic myocardium, a glucose/serum-deprived/hypoxia-induced apoptosis model (OGD) and an H_2_O_2_-induced apoptosis model were established as previously described [18]. For the OGD model, forty-eight hours after lentivirus infection, MSCs_Vec_ (MSCs infected with vector) or MSCs_FAIM_ (MSCs infected with murine FAIM) were washed with PBS and cultured with glucose-free DMEM under hypoxic conditions (0.3% O_2_) for an additional 12 h until they were harvested. An H_2_O_2_-induced apoptosis model was also used in this study. In brief, after transfection with lentivirus, MSCs were challenged with 1 mM H_2_O_2_ in glucose-free DMEM for 2 h until they were harvested.

### 2.5. TUNEL Assays

TUNEL assays were carried out using a TUNEL staining kit (#C1090, Beyotime, Shanghai, China). In brief, cells were fixed in 10% formalin and permeabilized with 0.5% Triton X-100, followed by incubation with TUNEL staining reagent at 37°C in the dark for 1 hour. Additionally, nuclei were stained with Hoechst 33258 (#H3569, Thermo Fisher, CA, USA). Fluorescence images were acquired, and the apoptotic rates were calculated as the percentages of TUNEL-positive cells.

### 2.6. Annexin V/Propidium Iodide (PI) Staining

Annexin V/PI staining was carried out using an Annexin V-APC/PI apoptosis kit (#70-AP107, Liankebio, Hangzhou, China). Briefly, the culture medium was collected, and cells were digested with trypsin. The two components were then mixed and centrifuged. Then, the supernatant was discarded, and 1× binding buffer was added to resuspend the MSCs. The single-cell suspensions were incubated with an Annexin V-APC and PI mixture for 15 min at room temperature and then quantified with an LSRFortessa flow cytometer. The apoptosis rate was calculated as the sum of the percentages of Annexin V^+^/PI^−^ cells and Annexin V^+^/PI^+^ cells.

### 2.7. RNA Analysis

Total RNA was isolated using TRIzol reagent (#15596-018, Invitrogen) and reverse-transcribed into cDNA with PrimeScript RT Master Mix (#RR036A, TaKaRa, Beijing, China). For qRT–PCR, cDNA was amplified using TB Green Premix Ex Taq™ (#RR420A, TaKaRa) on a LightCycler 480 system (Roche, USA). The mRNA expression levels were determined using the 2^−ΔCt^ method, and the results were normalized to *β*-actin. The primer sequences used for qRT–PCR are listed in Table 2.

### 2.8. Western Blotting

Whole-cell lysate was isolated using RIPA buffer (#P0013B, Beyotime) supplemented with a 1× protease and phosphatase inhibitor cocktail mixture (#78442, Thermo Fisher), and the total protein concentration was quantified with a BCA assay kit (#FD200, Fude Biological, Hangzhou, China). Proteins were separated by SDS–PAGE and then transferred onto PVDF membranes. After blocking with 5% skim milk (#LP0031B, Thermo Fisher), the membranes were incubated overnight at 4°C with the specific primary antibodies listed in Table 3, followed by HRP-linked secondary antibody incubation. Finally, the proteins were detected by an Amersham ImageQuant 800 Western blot imaging system using ECL Western blotting substrate (#FD8020, Fude Biological). The images were quantified using ImageQuant TL 8.2 analysis software, and the protein expression levels were normalized to the housekeeping protein GAPDH or *β*-actin.

### 2.9. Mouse MI Model and MSCs Transplantation

Animal breeding, maintenance, and procedures were performed in accordance with the Guide for the Care and Use of Laboratory Animals published by the NIH and approved by the Animal Use Committee of Zhejiang University.

A mouse MI model was established in 10- to 12-week-old male C57BL/6 J mice (23-26 g, Hangzhou Medical College Animal Research Center, Hangzhou, China) by left anterior descending coronary artery (LAD) ligation. Then, MSCs_FAIM_ or MSCs_Vec_ (1.5 × 10^5 cells in 20 *μ*l of DMEM per mouse) were immediately transplanted into the border zone of the ischemic area by intramyocardial injection. Control mice were injected with an equivalent volume of DMEM.

### 2.10. Echocardiography

Transthoracic echocardiography was performed to evaluate cardiac function on days 0, 3, 7, 14, and 28 after MI surgery. Mice were anesthetized with 2% isoflurane in 95% O_2_. Serial B-mode and M-mode images were obtained using a Vevo® 2100 ultrasound system (Visual Sonics, Canada), followed by cardiac function analysis using Vevo® LAB software (version 3.1.0).

### 2.11. Sirius Red Staining

Mice were euthanized by cervical dislocation after being injected with sodium pentobarbital. The hearts were harvested, fixed with 10% formalin, embedded in paraffin, and subsequently sectioned into 3 *μ*m slices. The slices were stained with Sirius Red (#SBJ-0294, SenBeiJia Biological, Nanjing, China). The images were quantitatively analyzed using ImageJ software (NIH, USA). The infarct size was calculated as follows: infarct size = [(endocardial + epicardial circumference of the infarct area)/(endocardial + epicardial circumference)] × 100%.

### 2.12. Immunofluorescence Staining

OCT-embedded hearts were sectioned in a cryostat at a thickness of 7 *μ*m. The slices were fixed with 10% formalin for 10 min, permeabilized with 0.5% Triton X-100 for 15 min, and blocked with 3% BSA (#B265993, Aladdin, Shanghai, China) for 30 min. Subsequently, overnight incubation was performed with the specific primary antibodies listed in Table 4, followed by incubation with Alexa Fluor-conjugated secondary antibody. Nuclei were stained with DAPI mounting medium (#H-1200-10, Vector, CA, USA).

### 2.13. Tube Formation Assays

Tube formation assays were conducted to evaluate the angiogenic potential of MSCs in response to different treatments. Human umbilical vein endothelial cells (HUVECs) were digested, counted, and resuspended in different conditioned media. Then, single-cell suspensions of the HUVECs were seeded into 96-well plates (1.2 × 10^4^ cells per well) coated with 50 *μ*l of solidified Matrigel (#354230, Corning, MA, USA) and cultured for 6 h under standard conditions. Images were captured with Leica DM IL LED microscopes, and the branch points and capillary lengths were quantified with ImageJ software using the angiogenesis analyzer plugin.

### 2.14. Cycloheximide (CHX) Chase Assay

To evaluate protein stability, MSCs were treated with 10 *μ*g/ml CHX (#S7418, Selleck) to arrest c-FLIP protein biosynthesis under hypoxic conditions for 0 h, 3 h, and 6 h. After the MSCs were harvested, c-FLIP protein expression was assessed by Western blotting.

### 2.15. Protein Degradation and Ubiquitination Assays

To examine protein degradation, MG132 (10 *μ*M, #HY-13259, MCE, Shanghai, China) and chloroquine (CQ, 50 nM, #HY-17589A, MCE) were used to inhibit the ubiquitin–proteasome and autophagy pathways, respectively. Cells were incubated with MG132 or CQ for the indicated times before being harvested, and c-FLIP protein expression was assessed by Western blotting.

For the ubiquitination assay, MSCs were treated with 10 *μ*M MG132 under hypoxic conditions for 6 h before being harvested for immunoprecipitation. The lysates were incubated with anti-FLIP antibodies (#56343, CST, MA, USA) or normal rabbit IgG (#2729, CST), bound to magnetic protein A beads (Bio-Rad), washed thoroughly, and eluted by boiling in 2× loading buffer, followed by Western blotting to examine the level of ubiquitin bound to the c-FLIP protein.

### 2.16. Immunoprecipitation

Immunoprecipitation was performed as previously described [19]. Briefly, MSCs were lysed with NP-40 cell lysis buffer (#P0013F, Beyotime) supplemented with a 1× protease phosphatase inhibitor cocktail mixture, 10 mM N-ethylmaleimide (NEM) (# HY-D0843, MCE), and 1 mM EDTA. In total, 10% of the lysates was kept as input, and the remaining samples were used for immunoprecipitation. Rabbit monoclonal anti-FLIP antibodies (1 : 100, #56343, CST) or normal rabbit IgG was incubated with magnetic protein A beads (#1614013, Bio-Rad) for 1 h at room temperature. The supernatant was discarded, and the bead–antibody complexes were washed with lysis buffer 3 times. Then, the bead–antibody complexes were incubated overnight at 4°C with the cell lysates mentioned above. The bead–antibody–protein immunocomplexes were washed 3 times with lysis buffer and then boiled in loading buffer with SDS at 98°C for 5 min to elute the proteins from the beads. Immunoblotting was performed to analyze the ubiquitin levels.

### 2.17. Statistical Analysis

Representative experiments were repeated at least three times, and the results are depicted as the mean ± standard deviation (SD). Statistical differences between two sets of data were analyzed by Student's *t* test, and multiple groups were compared using one-way ANOVA with Tukey's posttest. All statistical analyses and graphing were performed with GraphPad Prism 8 software. Differences for which *P* < 0.05 were considered statistically significant.

## 3. Results

### 3.1. FAIM Promoted the Survival of MSCs In Vitro and In Vivo

**Figure 1:**
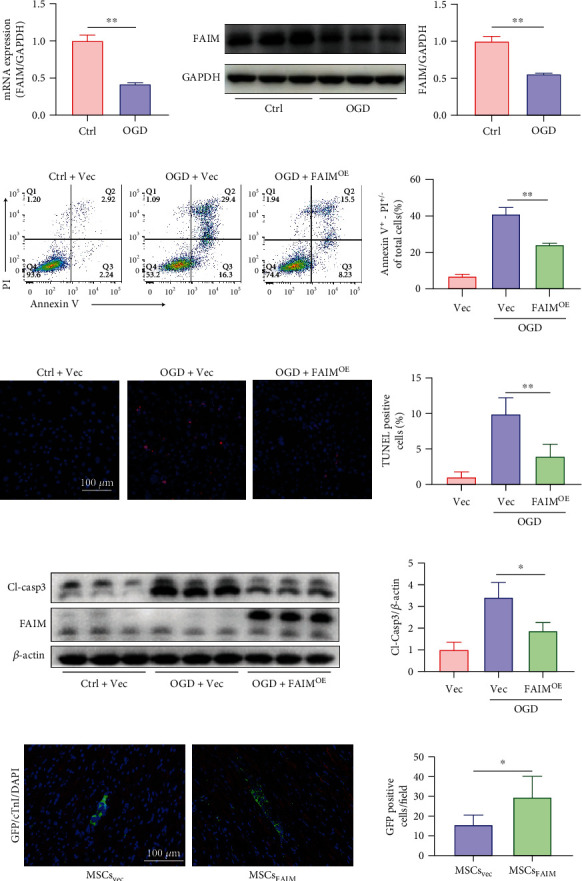
FAIM protected MSCs against apoptosis in vitro and in vivo. (a) MSCs were cultured for 12 h under OGD conditions or normoxic conditions, and the mRNA level of FAIM was determined by qPCR and normalized to GAPDH. (b) Immunoblot analysis and densitometric quantification of FAIM protein levels under OGD conditions or normoxic conditions. GAPDH served as a loading control. (c) Annexin V-APC/PI staining was performed, and flow cytometry was used to determine the apoptosis rate. The apoptosis rate was calculated as the sum of the percentages of Annexin V^+^/PI^−^ cells and Annexin V^+^/PI^+^ cells. (d) TUNEL staining after FAIM overexpression followed by OGD stimulation for 12 h (scale bar = 100 *μ*m). Quantitative results are shown on the right. Six visual fields were randomly chosen for each well; the apoptotic index was determined as the percentage of TUNEL-positive nuclei. (e) Cleaved caspase 3 protein expression after FAIM overexpression or vector infection followed by OGD stimulation. The results of densitometric quantitation are shown on the right. (f) Representative images showing GFP immunofluorescence (IF) staining 3 days after LAD ligation followed by MSC transplantation. GFP appears in green, cardiac troponin I (cTnI) in red, and nuclei in blue. (scale bar = 100 *μ*m). Quantitative results are shown on the right (*n* = 6 for the MSCs_Vec_ group and MSCs_FAIM_ group). Data are shown as the mean ± SD. ^∗^ denotes *P* < 0.05, ^∗∗^*P* < 0.01.

FAIM expression was found to be upregulated in SRT1720-pretreated MSCs, and FAIM knockdown abolished the protective effects of SRT1720 on MSCs in our previous study [20]. Thus, we speculated that FAIM may have an important effect on the apoptosis of transplanted MSCs. To explore this hypothesis, two cell apoptosis models were used in vitro to mimic ischemia or ROS generation in the MI microenvironment: the OGD model and the H_2_O_2_ model (Supplementary Figure S2). As shown in Figures 1(a) and 1(b), the mRNA and protein levels of FAIM were robustly decreased in the OGD model. The same reduction in FAIM protein expression was observed in response to H_2_O_2_ exposure (Supplementary Figure S3(a)). Thus, a FAIM overexpression experiment was carried out to determine the antiapoptotic effect of FAIM in the OGD model (Supplementary Figure S4(a)-4(b)), and the cell apoptosis rate was evaluated by an Annexin V-APC/PI assay. The FAIM^OE^ group showed a robust decrease in the percentage of apoptotic cells (Annexin V-positive) under OGD conditions compared with that of the vector group (Figure 1(c)), and this result was further supported by TUNEL staining (Figure 1(d)). Furthermore, the protein expression of cleaved caspase-3, the critical executioner of apoptosis, was examined by Western blotting. We found that FAIM overexpression significantly attenuated the activation of caspase-3 (Figure 1(e)). To explore the antiapoptotic effect of FAIM on MSCs in vivo, MSCs were marked with GFP and injected into the peri-infarct region immediately after LAD ligation. The MSCs retention rate was assessed by fluorescence microscopy 3 days after engraftment (Supplementary Figure S2). The immunofluorescence results showed that while no significant differences were observed between the two groups of cells (Supplementary Figure S4(a)), more FAIM-overexpressing MSCs (MSCs_FAIM_) than MSCs infected with vector (MSCs_Vec_) remained in the target tissue (Figure 1(f)). These results strongly suggested that FAIM inhibited caspase-3-dependent apoptosis under hypoxic conditions in vitro and in vivo.

### 3.2. FAIM-Overexpressing MSCs Improved Cardiac Function and Reduced Infarct Size

**Figure 2:**
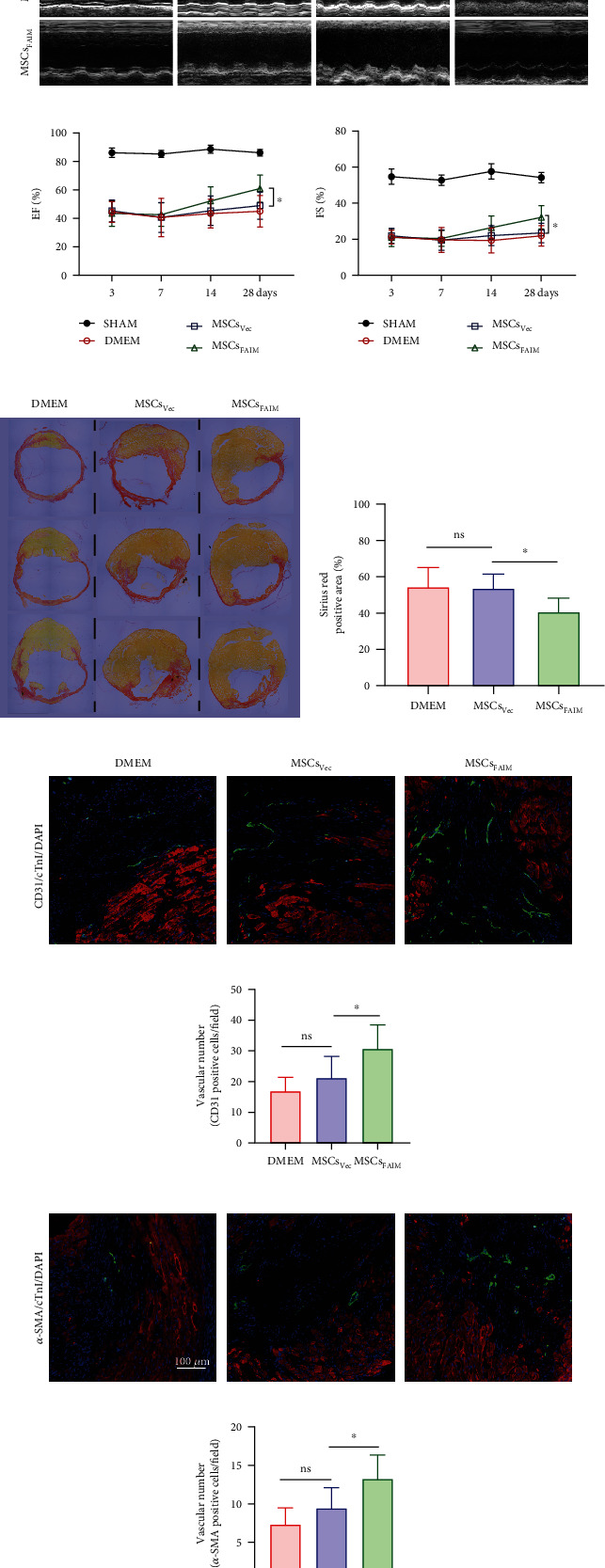
MSCs_FAIM_ improved cardiac function and reduced infarction size. (a) Representative echocardiographic images of the left ventricle in M-mode on days 3, 7, 14, and 28 after LAD ligation followed by MSCs engraftment. (b) Ejection fraction (EF) and (c) fractional shortening (FS) were quantified (*n* = 6 for the sham group, *n* = 7 for the DMEM and MSCs_Vec_ groups, and *n* = 8 for the MSCs_FAIM_ group). (d) Representative Sirius Red staining of ischemic hearts 28 days after MI. (e) Scar size was calculated as the sum of the ratio of the endocardial plus epicardial scar length relative to the total circumference (*n* = 7 for the DMEM and MSCs_Vec_ groups and *n* = 8 for the MSCs_FAIM_ group). (f–g) Representative images showing CD31 immunofluorescence staining in the peri-ischemic area 28 days after MI. CD31 appears in green, cardiac troponin I (cTnI) in red, and nuclei in blue (*scale* *bar* = 100 *μm*). Quantitative results are shown on the right (*n* = 7 for the DMEM and MSCs_Vec_ groups and *n* = 8 for the MSCs_FAIM_ group). (h–i) Representative images showing *α*-SMA IF staining. *α*-SMA appears in green; scale bar = 100 *μ*m. Quantitative results are shown on the right (*n* = 7 for the DMEM and MSCs_Vec_ groups and *n* = 8 for the MSCs_FAIM_ group). Vessels in the peri-ischemic area were counted in 5 randomly chosen fields. The data are shown as the mean ± SD. Ns indicates not significant; ^∗^ denotes *P* < 0.05.

To evaluate the effect of FAIM overexpression on the improvement in the therapeutic effect of MSCs, MSCs_Vec_ and MSCs_FAIM_ were transplanted into the peri-ischemic area immediately after MI model construction, and an equivalent volume of DMEM was injected into the ischemic hearts in the control group. Transthoracic echocardiography was conducted on days 3, 7, 14, and 28 after cell transplantation to evaluate cardiac function, and Sirius Red staining was conducted 28 days after MI to examine the fibrotic area (Supplementary Figure S2). The echocardiography data revealed that compared with the DMEM and MSCs_Vec_ groups, cardiac function after MI was significantly improved in the MSCs_FAIM_ group (*n* = 6 for the sham group, *n* = 7 for the DMEM and MSCs_Vec_ groups, and *n* = 8 for the MSCs_FAIM_ group) (Figures 2(a)–2(c)). Histological analysis also confirmed these results, and a considerable reduction in infarct scarring was observed in the MSCs_FAIM_ group on day 28 after cell transplantation by Sirius Red staining (Figures 2(d)–2(e)).

Accumulating evidence indicates that the effects of MSCs are principally attributed to their abundant paracrine functions, which result in angiogenesis [21]. Thus, we hypothesized that the increased retention of MSCs_FAIM_ would increase angiogenesis. To validate this hypothesis, the capillary density and microvasculature in the peri-infarct area were measured. Compared with those in the DMEM group and MSCs_Vec_ group, increased numbers of endothelial cells (CD31-positive) and vascular smooth muscle cells (*α*-SMA-positive) was observed in the MSCs_FAIM_ group 28 days after MSCs engraftment (Figures 2(f)–2(i)). Furthermore, an in vitro tube formation assay was conducted to evaluate the proangiogenic capacity of conditioned medium from MSCs. The supernatants of MSCs_Vec_ and MSCs_FAIM_ cultured under normoxic conditions and hypoxic conditions were used to culture HUVECs. No clear differences were observed between the MSCs_FAIM_ group and the MSCs_Vec_ group under normoxic conditions; however, culture with the supernatants of hypoxia-treated MSCs_Vec_ led to a significant decrease in angiogenesis, and FAIM overexpression rescued this reduction (Supplementary Figure S6). Taken together, these results suggested that MSCs_FAIM_ more effectively promoted angiogenesis, improved cardiac function, and limited the fibrotic area than did the MSCs_Vec_.

### 3.3. FAIM Regulates the Expression of c-FLIP in MSCs under OGD Conditions

**Figure 3:**
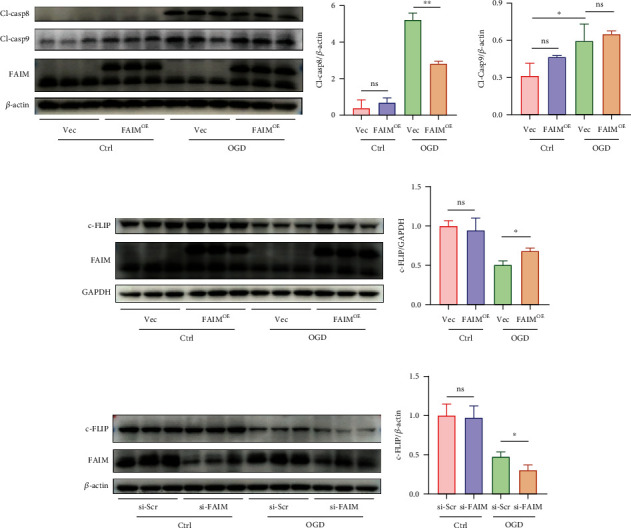
FAIM regulated the expression of c-FLIP in MSCs under OGD conditions. (a–b) Immunoblots and densitometric quantitation of cleaved caspase-8 and cleaved caspase-9 protein levels in MSCs_Vec_ and MSCs_FAIM_ under OGD conditions. (c–d) Immunoblots and densitometric quantitation of c-FLIP protein levels in MSCs_Vec_ and MSCs_FAIM_ under OGD conditions. (e–f) c-FLIP protein levels after siRNA-mediated knockdown of FAIM or scrambled siRNA administration followed by OGD stimulation for 12 h. The results of densitometric quantification are shown on the right. The data are shown as the mean ± SD. Ns indicates not significant; ^∗^ denotes *P* < 0.05, ^∗∗^*P* < 0.01.

The extrinsic apoptosis pathway, which is mediated by DRs, and the intrinsic apoptosis pathway, which is mediated by mitochondria, are the two main pathways through which apoptosis is induced. Caspase-8 and caspase-9 are the initiating caspases in these two pathways [22]. Several studies have demonstrated that FAIM strongly inhibits DR signaling and Fas-mediated apoptosis [12]; however, the specific role of FAIM in hypoxia-induced apoptosis is poorly understood. Thus, the protein expression of cleaved caspase-8 and cleaved caspase-9 was determined. As expected, FAIM overexpression reduced the cleavage of caspase-8 but had no significant effect on caspase-9 activation (Figures 3(a) and 3(b)). c-FLIP regulates the activation of caspase-8, and FAIM-deficient hepatocytes exhibit decreased c-FLIP protein expression [17]. Therefore, to test whether c-FLIP is involved in hypoxia-induced apoptosis, and whether FAIM can regulate c-FLIP expression, the protein expression level of c-FLIP was examined after the knockdown or overexpression of FAIM and culture under hypoxic conditions by Western blotting. c-FLIP protein expression was notably decreased in the context of hypoxia, and FAIM overexpression blocked this reduction (Figures 3(c) and 3(d)). Not surprisingly, c-FLIP expression notably declined after FAIM knockdown (Figures 3(e) and 3(f)).

### 3.4. c-FLIP Is Required for the Antiapoptotic Effect of FAIM under OGD Conditions

**Figure 4:**
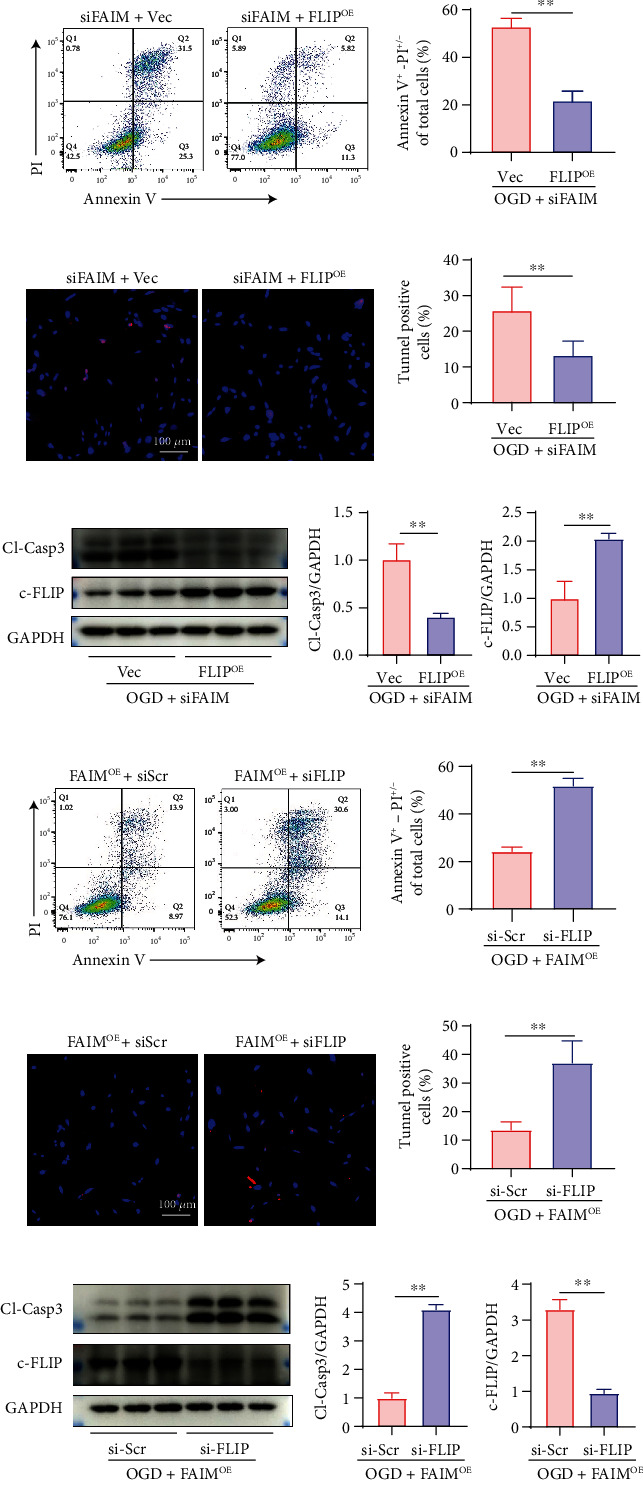
c-FLIP is required for the antiapoptotic effect of FAIM. MSCs_Vec_ and MSCs_FLIP_ were transfected with si-FAIM and exposed to OGD. (a–b) Annexin V-APC/PI staining was performed, and flow cytometry was used to determine the apoptosis rate. (c–d) TUNEL staining of MSCs_Vec_ and MSCs_FLIP_ after FAIM knockdown followed by OGD treatment for 12 h. (e–f) Cleaved caspase 3 protein expression was detected by Western blotting. (g–h) Annexin V-APC/PI staining of MSCs_FAIM_ transfected with si-FLIP or si-Scr under OGD conditions. (i–j) TUNEL staining of MSCs_FAIM_ after FLIP knockdown or control treatment followed by OGD treatment for 12 h. (k–l) Cleaved caspase 3 protein expression was measured by Western blotting. The data are shown as the mean ± SD. Ns indicates not significant; ^∗∗^denotes *P* < 0.01.

The relationship between c-FLIP and FAIM was further confirmed. MSCs overexpressing c-FLIP (MSCs_FLIP_) were generated by lentivirus infection, whereas MSCs infected with empty lentivirus labeled with GFP (GFP-Vec) served as the negative control (MSCs_Vec_). The efficiency of c-FLIP overexpression was detected by Western blotting (Supplementary Figure S4(d)). FAIM was silenced by siRNA in both MSCs_Vec_ and MSCs_FLIP_, which was followed by OGD exposure. Upon FAIM silencing, c-FLIP overexpression reduced the percentage of apoptotic MSCs, as shown by Annexin V/PI staining and TUNEL assays (Figures 4(a)–4(d)). Western blotting further confirmed that the protein levels of cleaved caspase-3 were significantly decreased by c-FLIP overexpression (Figures 4(e) and 4(f)). Furthermore, MSCs_FAIM_ were transfected with either siRNA specific for c-FLIP (si-FLIP) or scrambled siRNA (si-Scr), followed by OGD challenge. Under OGD conditions, c-FLIP silencing significantly elevated the percentage of Annexin V-positive MSCs_FAIM_ (Figures 4(g) and 4(h)) and the protein expression of cleaved caspase-3 (Figures 4(k) and 4(l)), and the results were also confirmed by TUNEL staining (Figures 4(i) and 4(j)). These results indicated that the antiapoptotic effect of FAIM under OGD conditions was at least partially dependent on c-FLIP.

### 3.5. FAIM Stabilizes c-FLIP Protein in MSCs by Inhibiting Its Ubiquitination

**Figure 5:**
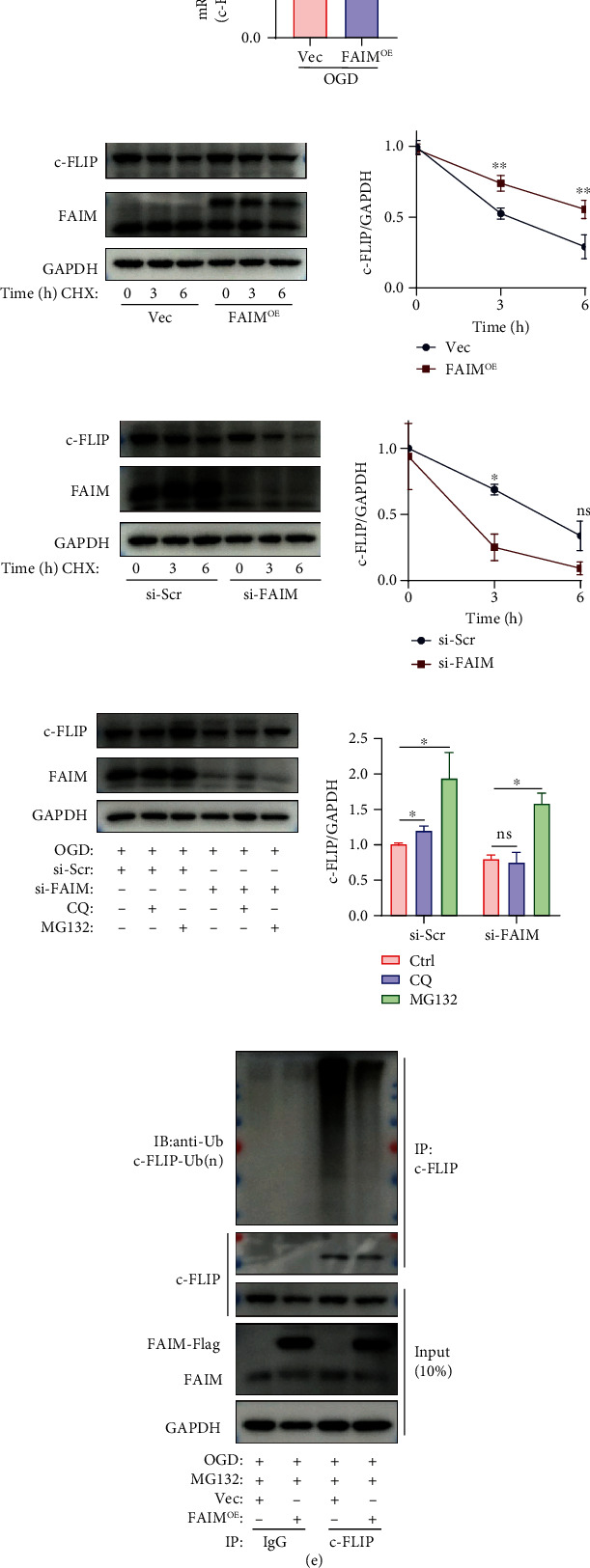
FAIM stabilizes the c-FLIP protein in MSCs by inhibiting its ubiquitination. (a) mRNA expression of c-FLIP in MSCs_Vec_ and MSCs_FAIM_ challenged with OGD. (b) Representative immunoblots showing c-FLIP protein expression in MSCs_Vec_ and MSCs_FAIM_ treated with 10 *μ*M CHX for the indicated times under OGD conditions. The results of densitometric quantitation are shown on the right, and the line chart shows c-FLIP protein levels (normalized to GAPDH) as a percentage of the c-FLIP protein expression of MSCs_Vec_ at 0 h (*n* = 3). (c) Representative immunoblots showing c-FLIP protein levels in MSCs_si-scr_ and MSCs_si-FAIM_ treated with 10 *μ*M CHX for the indicated times under OGD conditions. The results of densitometric quantitation are shown on the right (*n* = 3). (d) Representative Western blotting results showing c-FLIP protein expression after siRNA-mediated knockdown of FAIM or scrambled siRNA administration followed by treatment with chloroquine (CQ, 50 nM) or MG132 (10 *μ*M) under OGD conditions. The results of densitometric quantitation are shown below (*n* = 3). (e) MSCs_Vec_ and MSCs_FAIM_ were treated with MG132 (10 *μ*M) under OGD conditions for 6 h. Whole-cell lysates were immunoprecipitated with anti-FLIP antibodies, followed by immunoblotting with anti-ubiquitin antibodies. The input (10% of the total) was analyzed by Western blotting using anti-FLIP and anti-FAIM antibodies. The data are shown as the mean ± SD. Ns indicates not significant; ^∗^ denotes *P* < 0.05; ^∗∗^*P* < 0.01.

To determine the underlying mechanisms, we examined the mRNA level of c-FLIP and found that FAIM overexpression did not alter the transcript expression of c-FLIP (Figure 5(a)). To reveal whether FAIM posttranscriptionally regulates the expression of c-FLIP, a CHX chase assay was performed to test the change in protein stability of c-FLIP in response to FAIM knockdown and overexpression. We found that FAIM overexpression significantly inhibited the decrease in c-FLIP protein expression (Figure 5(b)), but FAIM knockdown facilitated a reduction in c-FLIP expression in MSCs (Figure 5(c)). These data suggested that the degradation of c-FLIP could be impaired by FAIM.

Autophagy and proteasome-dependent proteolysis are the two main ways by which protein degradation is regulated. Consequently, hypoxia-challenged MSCs were treated with CQ to inhibit the autophagy–lysosome pathway and MG132 to impede the proteasome-dependent pathway. In comparison with that in response to DMSO, the expression of c-FLIP was strongly restored by MG132 but barely affected by CQ (Figure 5(d)). Collectively, these data showed that FAIM upregulates c-FLIP protein expression largely by inhibiting its proteasome-dependent degradation. To further investigate the ubiquitination of c-FLIP, we treated OGD-challenged MSCs with MG132 to arrest the degradation of c-FLIP without affecting its ubiquitination 6 h before harvesting for analysis. We found that c-FLIP was ubiquitinated under hypoxic conditions, and FAIM overexpression markedly decreased the ubiquitination level of c-FLIP (Figure 5(e)). This result complements our previous finding showing that FAIM modulates c-FLIP expression by inhibiting its ubiquitination–proteasome-dependent proteolysis.

### 3.6. FAIM Reduces c-FLIP Degradation by Blocking JNK Activation

**Figure 6:**
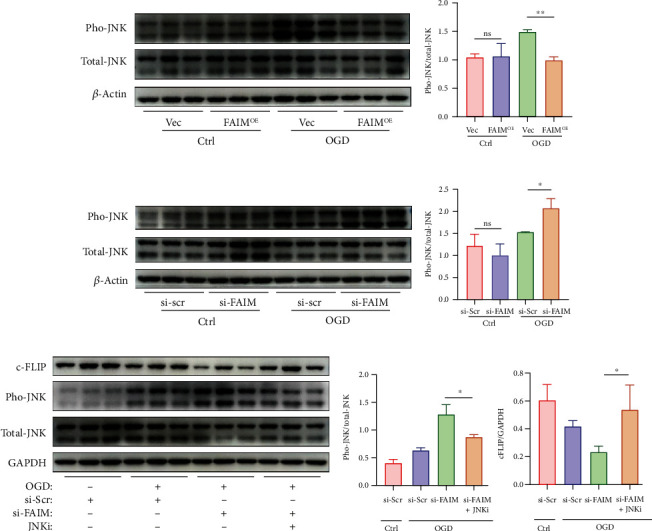
FAIM reduces c-FLIP degradation by blocking JNK activation. (a–b) Immunoblots and densitometric quantitation of phosphorylated JNK and total JNK protein expression in MSCs_Vec_ and MSCs_FAIM_ under OGD conditions. (c–d) Western blotting and densitometric quantitation of phosphorylated JNK and total JNK protein expression in MSCs_si-scr_ and MSCs_si-FAIM_ under OGD conditions. (e–f) Phosphorylated JNK, total JNK, and c-FLIP protein expression after siRNA-mediated knockdown of FAIM or scrambled siRNA administration followed by treatment with SP600125 (a JNK inhibitor, 10 *μ*M) or DMSO under OGD conditions. The data are shown as the mean ± SD. Ns indicates not significant; ^∗^ denotes *P* < 0.05; ^∗∗^*P* < 0.01.

Phosphorylated JNK has been reported to be upregulated during apoptosis [23] and has also been reported to participate in the degradation of c-FLIP [24]. We hypothesized that the JNK pathway is involved in hypoxia-induced apoptosis, and that FAIM attenuates the degradation of c-FLIP by inhibiting the phosphorylation of JNK. To validate this hypothesis, phosphorylated JNK was assessed by Western blotting. As expected, we found that JNK phosphorylation was strongly inhibited by FAIM in hypoxic MSCs (Figures 6(a) and 6(b)), and FAIM silencing increased this phosphorylation (Figures 6(c) and 6(d)). We further treated FAIM-deficient MSCs with SP600125, a specific JNK inhibitor, and verify its impact on c-FLIP protein expression. We found that SP600125 upregulated c-FLIP protein expression (Figures 6(e) and 6(f)), verifying that FAIM inhibits c-FLIP degradation mainly by blocking JNK activation.

**Figure 7:**
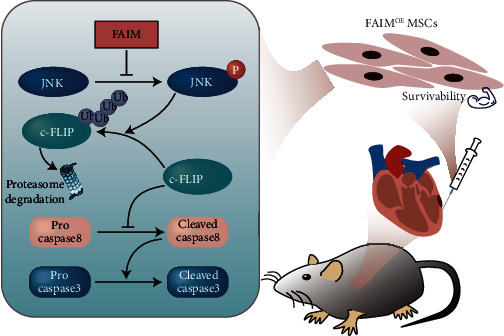
Graphical abstract: Schematic diagram of the proposed mechanism; schematic diagram of the proposed mechanisms by which FAIM impairs the JNK-mediated, ubiquitination–proteasome-dependent degradation of c-FLIP, thereby effectively promoting MSC survival and ultimately ameliorating cardiac function and improving the prognosis of MI in vivo.

Taken together, our results provide evidence that FAIM inhibits MSC apoptosis by inhibiting the ubiquitination-dependent degradation of c-FLIP, which is mediated by JNK inactivation, thereby improving the efficacy of MSC transplantation (Figure 7).

## 4. Discussion

In this study, we showed that FAIM was downregulated in MSCs under ischemia-like conditions, including hypoxia and oxidative stress, in vitro. Consequently, we overexpressed FAIM in MSCs and found that FAIM augmentation protected MSCs from apoptosis in a harsh microenvironment. Similarly, we observed improved MSCs survival in a mouse MI model. Furthermore, improvements in cardiac function and a reduced infarction size were detected in the MSCs_FAIM_ group 28 days after transplantation, accompanied by increased angiogenesis, suggesting that FAIM augmentation improved the efficacy of MSCs transplantation in a mouse MI model. Mechanistically, our findings demonstrated that the antiapoptotic effect of FAIM is largely dependent on c-FLIP, which suppresses the activation of caspase-8. Next, we found that FAIM had no effect on c-FLIP transcription but altered the protein degradation of c-FLIP. Further experiments demonstrated that FAIM protects c-FLIP from ubiquitination–proteasome-dependent degradation, which is mediated by impaired JNK phosphorylation. These data provide a novel approach to improve the effects of stem cells engraftment on cardiovascular indications.

MI poses a grave threat to the health of elderly individuals, and the loss of cardiomyocytes after MI is the most important cause of poor prognosis [25]. Therefore, remuscularization of the heart is the major focus in this field, and countless efforts have been made to find solutions, including promoting the regeneration of cardiomyocytes, transplanting induced pluripotent stem cell (iPSC)-derived cardiomyocytes, exogenous engrafting of stem cells or their derived exosomes, and the application of novel functional materials such as engineered heart tissues and hydrogels [26–29]. It has been demonstrated that within one week of birth, the hearts of neonatal mice exhibit regenerative activity, and subsequent studies showed that regenerative activity also occurred in neonatal pigs [30, 31]. Exploring the mechanism of cardiomyocyte regeneration and implementing treatments have become important research directions. A recent study reported that cardiac-specific knockout of PTEN facilitated the proliferation and renewal of cardiomyocytes [32]. Moreover, Salvador-siRNA delivered by adeno-associated virus 9 promoted the proliferation of cardiomyocytes after ischemia and reperfusion injury by inhibiting the Hippo pathway, which repressed cardiomyocyte proliferation after MI [33]. However, these studies are still in their early stages, and it could take decades to achieve clinical translational applications. In addition, engineering materials have provided some new ideas for the treatment of MI [34]. Patches were used to provide mechanical support to improve outcomes in the early days after MI, and subsequent studies reported the development of patches equipped with anti-inflammatory activity, but they could not compensate for the loss of cardiomyocytes [29]. However, the unique physical and electrical properties of the heart make it difficult for simple bioengineering materials to be of great use, and those engineering materials are often functionalized through synergistic application with stem cells or iPSC-derived cardiomyocytes [35, 36]. Taken together, these results highlight the importance of cell-based therapies for MI treatment.

Tremendous improvements in cell-based therapies for MI have been made in recent decades, and these therapies aim to produce a sufficient number of cardiomyocytes to compensate for the loss of viable cardiomyocytes after MI [37]. A variety of cells are used in the treatment of MI, such as human iPSCs (hiPSCs), human embryonic stem cells (hESCs), and the widely used hMSCs [27, 38]. Generating cardiomyocytes from hiPSCs are no longer difficult [39], and this strategy has been applied in porcine MI models [40, 41]. However, there are still many challenges before iPSC-derived cardiomyocytes move to early trials, such as arrhythmias that can result from the injection of cardiomyocytes, the long-term immunosuppressant therapy required due to allogeneic-derived iPSCs, and the purity of the large number of iPSC-derived cardiomyocytes during induced proliferation and differentiation [26]. MSCs are ideal candidates for large-scale application in the clinical treatment of MI. Recent studies have shown that the therapeutic effects of MSCs are mainly exerted through their robust paracrine effects [21, 42], which promote the proliferation and migration of endothelial cells and ultimately improve cardiac angiogenesis and left ventricular function [43]. However, the harsh microenvironment formed by conditions including ischemia, hypoxia, oxidative stress, and inflammation causes massive cell death. A large proportion of engrafted stem cells were reported to perish within 24 h after transplantation [44, 45], making strategies that prolong MSCs lifespan an attractive way to improve the efficacy of MSCs [46]. Our previous study showed that pretreatment with SRT1720 improved the efficacy of MSCs in rodent and nonhuman primate MI models by prolonging MSCs survival [20, 47]. Consistent with these findings, our results showed that FAIM augmentation improved implanted MSCs survival and ultimately promoted neovascularization and ameliorated cardiac function.

Two splicing variants of FAIM have been described; the longer variant, FAIM-L, which is exclusively expressed in neural tissue, and the ubiquitously expressed shorter variant, known as FAIM-S [10]. FAIM-L exerts antiapoptotic effects by suppressing TCR signaling or DR signaling [11, 12]. However, to the best of our knowledge, this is the first study to reveal the protective effect of FAIM-L against apoptosis induced by hypoxia. Previous studies reported that c-FLIP expression was perturbed in FAIM-depleted cells [17]. Consistent with these findings, our results showed that the survival effect of FAIM was mainly achieved by restoring c-FLIP expression. However, to date, the effects of FAIM on c-FLIP expression are not fully understood. FAIM has been reported to exert its biological effects by reducing ubiquitination; FAIM was found to reduce the ubiquitination and degradation of XIAP via direct interaction [48], FAIM knockout led to the presentation of ubiquitinated aggregates in the retina [49], and FAIM protects glutaminase C from ubiquitination and induces cancer cell proliferation in lung adenocarcinoma [14]. Here, we found that FAIM could maintain c-FLIP stabilization under hypoxic conditions by protecting c-FLIP from ubiquitination and degradation, thus protecting MSCs from apoptosis.

c-FLIP, a strong regulator of caspase-8 activation due to the pseudocaspase domain at its C-terminus [50], participates in various cell death pathways, including apoptosis, necroptosis, and autophagy [51, 52]. Recent studies have shown that c-FLIP prevents cells from undergoing apoptosis by impairing p53-mediated PUMA upregulation and caspase-8 activation [53]. However, the functions of c-FLIP are contradictory and depend on its abundance in the death-inducing signaling complex (DISC) [15], which is assembled from Fas, FADD, procaspase 8, and c-FLIP [54]. Low concentrations of c-FLIP in the DISC can cause the formation of a procaspase-8/c-FLIP heterodimer and promote caspase-8 activation [55]. On the other hand, c-FLIP at a high concentration competitively suppresses the assembly of procaspase-8 into the DISC and thus reduces the activation of caspase-8 [56]. In the present study, we demonstrated that high protein expression of c-FLIP inhibited the activation of caspase-8; however, the caspase-8 activation status under low c-FLIP expression levels was not examined because we could not confirm whether the cleavage of procaspase-8 caused by the decrease in c-FLIP protein expression was due to a decrease in inhibition or an increase in activation. Regardless, this does not affect our conclusion that FAIM overexpression reduces MSCs apoptosis by ameliorating the decreased expression of c-FLIP under OGD conditions. In addition, accumulating evidence suggests that c-FLIP is a short-lived protein whose half-life is approximately 3 h [24]. Some studies have shown that the degradation of c-FLIP occurs mainly through the ubiquitin–proteasome pathway [57], and JNK activity may be a critical regulator of c-FLIP protein stability [24]. In agreement with previous findings, we revealed that the protein stability of c-FLIP is closely related to the presence of FAIM, and subsequent experiments showed that FAIM regulates c-FLIP protein stability by impairing JNK phosphorylation, which expands the current knowledge of the protein stability of c-FLIP.

There are some limitations of this study. First, our experimental setting could not completely mimic true clinical conditions. MSCs were injected into the peri-infarct zones immediately after LAD ligation, which is unrealistic in the real world. Second, unbiased analysis was not utilized in this study, and mass spectrometry-based proteomics analysis may be a more accurate way to determine the most predominant protein that regulates the antiapoptotic effects of FAIM in our model. Third, the mechanism by which FAIM regulates JNK activation was not explored in this work.

## 5. Conclusion

In conclusion, our study demonstrated that FAIM augmentation could effectively promote MSCs survival in vivo and ultimately ameliorate cardiac function and improve the prognosis of MI, providing a novel approach to improve the efficacy of MSC-based therapy.

## Figures and Tables

**Table 1:** siRNA sequences.

siRNA name		Sequence (5′ − >3′)
si-FAIM	Sense	GGAUUAUCCAUACCCUCAUTT
	Antisense	AUGAGGGUAUGGAUAAUCCTT
si-c-FLIP	Sense	CAAGUAUGGCCCAACAUCATT
	Antisense	UGAUGUUGGGCCAUACUUGTT
si-scramble	Sense	UUCUCCGAACGUGUCACGUTT
	Antisense	ACGUGACACGUUCGGAGAATT

**Table 2:** qRT–PCR primer sequences.

Target gene	Primer name	Sequence (5′ − >3′)
*β*-actin	Forward primer	GGTGGGAATGGGTCAGAAGG
	Reverse primer	GTACATGGCTGGGGTGTTGA
FAIM	Forward primer	ATGGGACCACATCAGGCAAG
	Reverse primer	TCCAGCGTGTACTCGTATGC
c-FLIP	Forward primer	ACACAGGCAGAGGCAAGATA
	Reverse primer	TGGCTCTTTACTTCGCCCATT

**Table 3:** Antibodies used for immunoblotting.

Antibody	Species	Dilution	Catalog no.	Company
Anti-*β*-actin	Mouse	1/5000	#KC-5A08	KangCheng biotech
Anti-GAPDH	Mouse	1/5000	#KC-5G5	KangCheng biotech
Anti-FAIM	Rabbit	1/1000	#PA5-29200	Thermo fisher
Anti-cleaved caspase-3	Rabbit	1/1000	#9661	CST
Anti-cleaved caspase-8	Rabbit	1/1000	#8592	CST
Anti-cleaved caspase-9	Rabbit	1/1000	#9509	CST
Anti-FLIP (D5J1E)	Rabbit	1/1000	#56343	CST
Anti-SAPK/JNK	Rabbit	1/1000	#9252	CST
Anti-phospho-SAPK/JNK	Rabbit	1/1000	#4668	CST
HRP-linked anti-ubiquitin	Mouse	1/1000	#14049	CST
HRP-linked anti-mouse	Horse	1/3000	#7076	CST
HRP-linked anti-rabbit	Goat	1/3000	#7074	CST
HRP-linked anti-rabbit (light-chain specific)	Mouse	1/3000	#93702	CST

**Table 4:** Antibodies used for immunofluorescence.

Antibody	Species	Dilution	Catalog no.	Company
Anti-GFP	Rabbit	1/200	#ab290	Abcam
Anti-CD31	Rat	1/300	#550274	BD
Anti-*α*-SMA	Rabbit	1/200	#19245	CST
Anti-cTnI	Goat	1/300	#ab56357	Abcam
Anti-Rabbit-488	Donkey	1/300	#ab150073	Abcam
Anti-Goat-555	Donkey	1/300	#ab150134	Abcam
Anti-Rat-488	Donkey	1/300	#ab150153	Abcam

## Data Availability

The datasets used and/or analyzed to support the findings of this study are included within the article and supplementary information files.

## Abbreviations

MSCs: Mesenchymal stem cells
OGD: Oxygen, serum, and glucose deprivation
MI: Myocardial infarction.
LAD: Left anterior descending coronary artery
FAIM: Fas apoptosis inhibitory molecule
c-FLIP: Cellular-FLICE inhibitory protein
MSCs_Vec_: MSCs infected with vector
MSCs_FAIM_: MSCs infected with FAIM
MSCs_FLIP_: MSCs infected with c-FLIP
FADD: Fas-associated via death domain
DR: Death receptor
CHX: Cycloheximide.

## Supplementary Materials

Supplementary Materials Supplementary Figure S1: characterization of mesenchymal stem cells (MSCs). Supplementary Figure S2: schematic of the in vitro and in vivo experiments. Supplementary Figure S3: FAIM protected MSCs against H_2_O_2_-induced apoptosis in vitro. Supplementary Figure S4: identification of FAIM overexpression lentivirus and FAIM siRNA. Supplementary Figure S5: FAIM knockdown facilitated MSCs apoptosis. Supplementary Figure S6: FAIM overexpression reversed the decrease in the proangiogenic capacity of conditioned medium from MSCs induced by OGD.
