# Supplementary material for: FAIM Enhances the Efficacy of Mesenchymal Stem Cell Transplantation by Inhibiting JNK-Induced c-FLIP Ubiquitination and Degradation
Source: Stem Cells Int. 2022 Sep 30;2022:3705637. doi: 10.1155/2022/3705637 (PMC9553537; doi:10.1155/2022/3705637)
Supplement: Supplementary Materials — Supplementary Figure S1: characterization of mesenchymal stem cells (MSCs). Supplementary Figure S2: schematic of the in vitro and in vivo experiments. Supplementary Figure S3: FAIM protected MSCs against H2O2-induced apoptosis in vitro. Supplementary Figure S4: identification of FAIM overexpression lentivirus and FAIM siRNA. Supplementary Figure S5: FAIM knockdown facilitated MSCs apoptosis. Supplementary Figure S6: FAIM overexpression reversed the decrease in the proangiogenic capacity of conditioned medium from MSCs induced by OGD. [file 3705637.f1.docx]

**FAIM enhances the efficacy of mesenchymal stem cell transplantation by inhibiting JNK-induced c-FLIP ubiquitination and degradation**

**Supplementary Figures and Figure Legends**: page 1

**
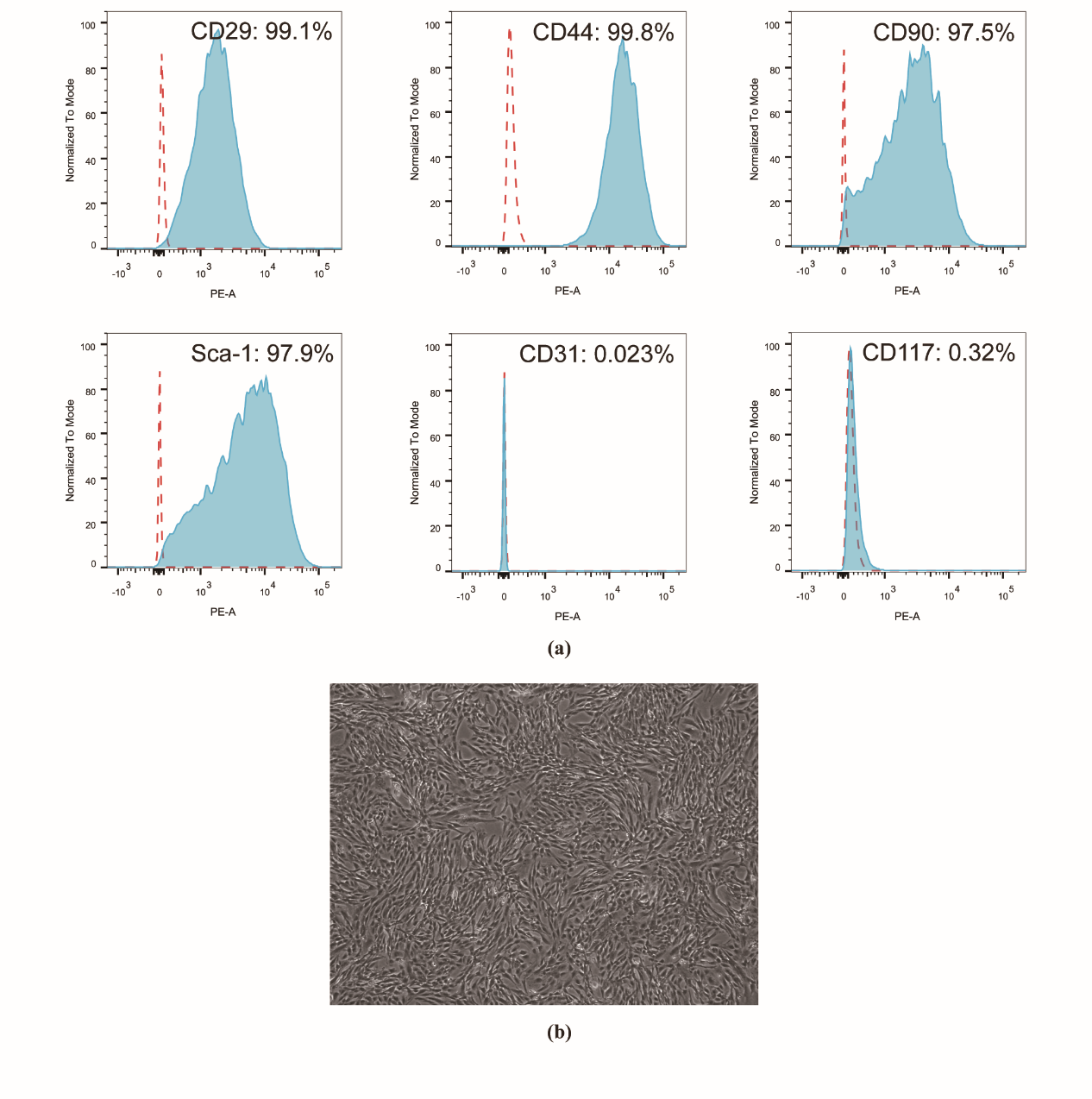
**

**Supplementary Figure S1** **Characterization of mesenchymal stem cells (MSCs)**

(a) Characterization of MSCs by flow cytometry. MSCs were considered to have positive expression of CD29, CD44, CD90 and Sca-1 and negative expression of CD31 and CD117. (b) Cell morphology observed with a microscope. MSCs were fusiform and arranged in a swirling pattern.


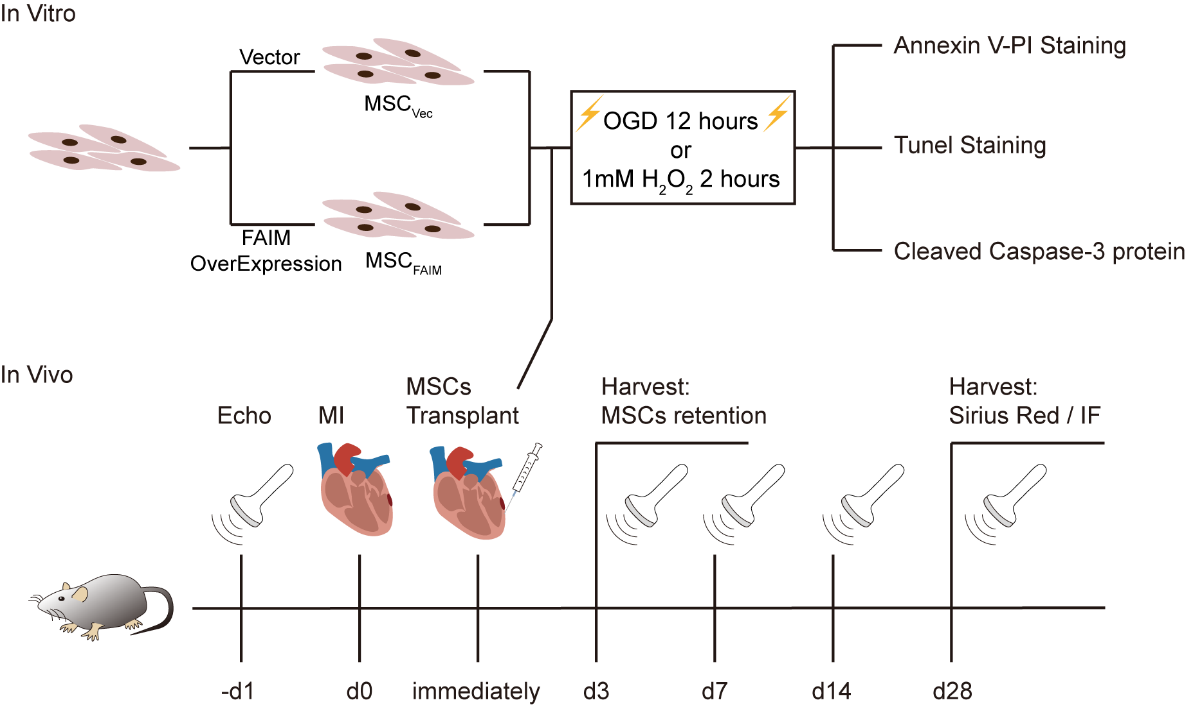
**Supplementary Figure S2**

Schematic of the in vitro and in vivo experiments.


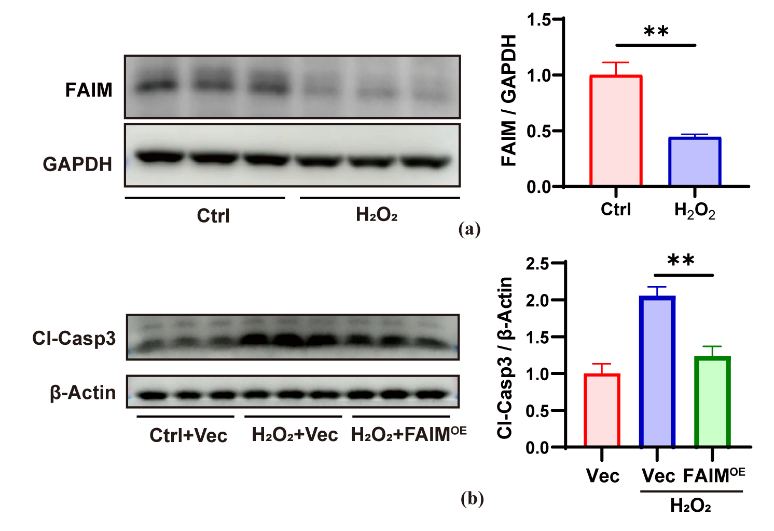


**Supplementary Figure S3 FAIM protected MSCs against H_2_O_2_ induced apoptosis in vitro**

(a) Immunoblot and densitometric quantification of FAIM protein levels in MSCs cultured with 1 mM H_2_O_2_ for 2 h. GAPDH served as a loading control. (b) Cleaved caspase-3 protein expression after FAIM overexpression or vehicle administration followed by 1 mM H_2_O_2_ treatment. β-Actin served as a loading control. The data are shown as the mean ± SD, ** denotes P < 0.01.


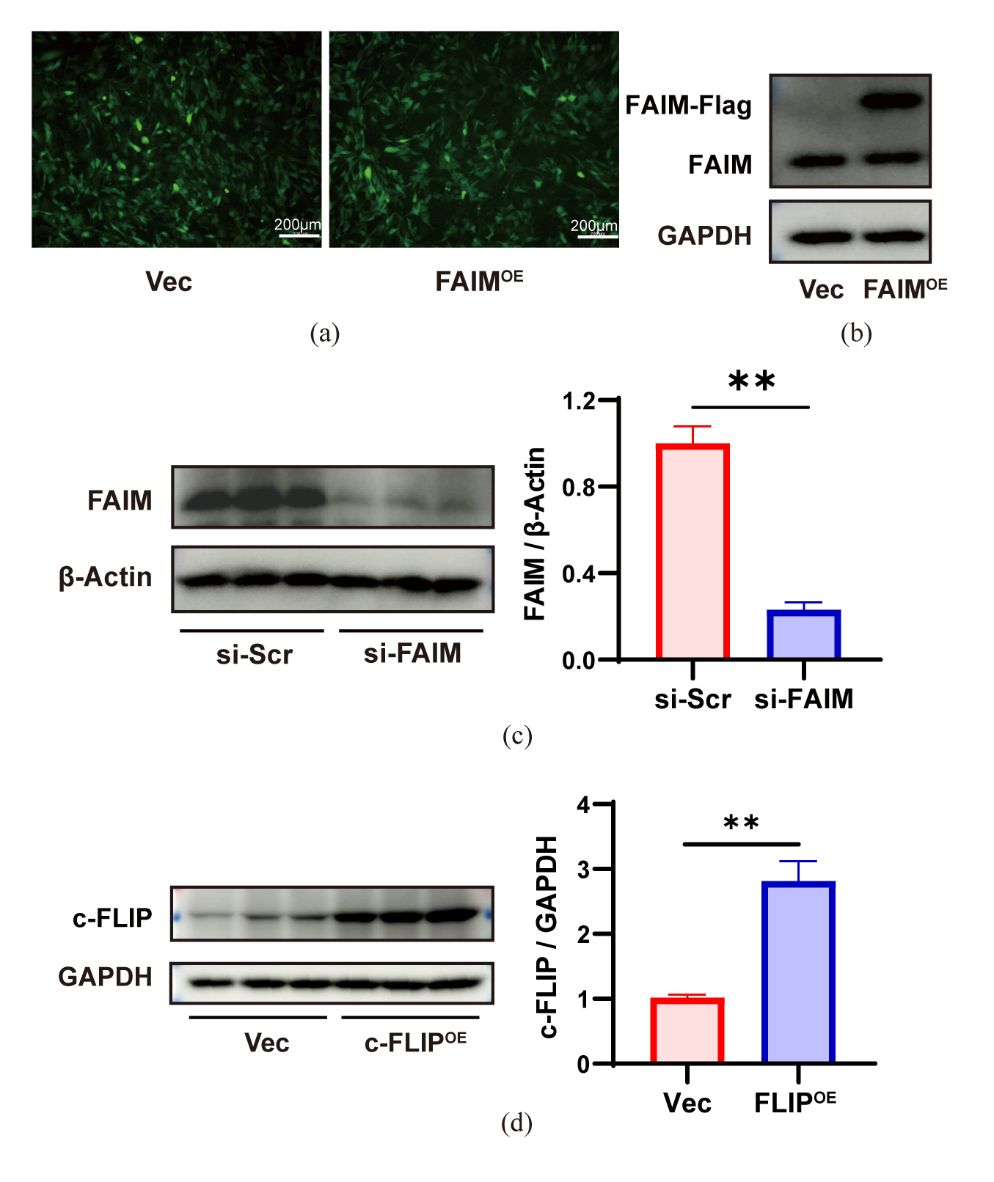


**Supplementary Figure S4** **Identification of FAIM overexpression lentivirus and FAIM siRNA**

(a) Representative images showing GFP immunofluorescence (IF) 2 days after FAIM-overexpression lentivirus infection (scale bar = 200 μm). (b) Immunoblot showing FAIM protein levels in MSCs 2 days after lentivirus infection. (c) Immunoblot showing FAIM protein levels in MSCs 2 days after siRNA-mediated knockdown of FAIM or scrambled siRNA administration. (d) Immunoblot showing c-FLIP protein levels in MSCs 2 days after infected with c-FLIP overexpression lentivirus. The data are shown as the mean ± SD, ** denotes P < 0.01.


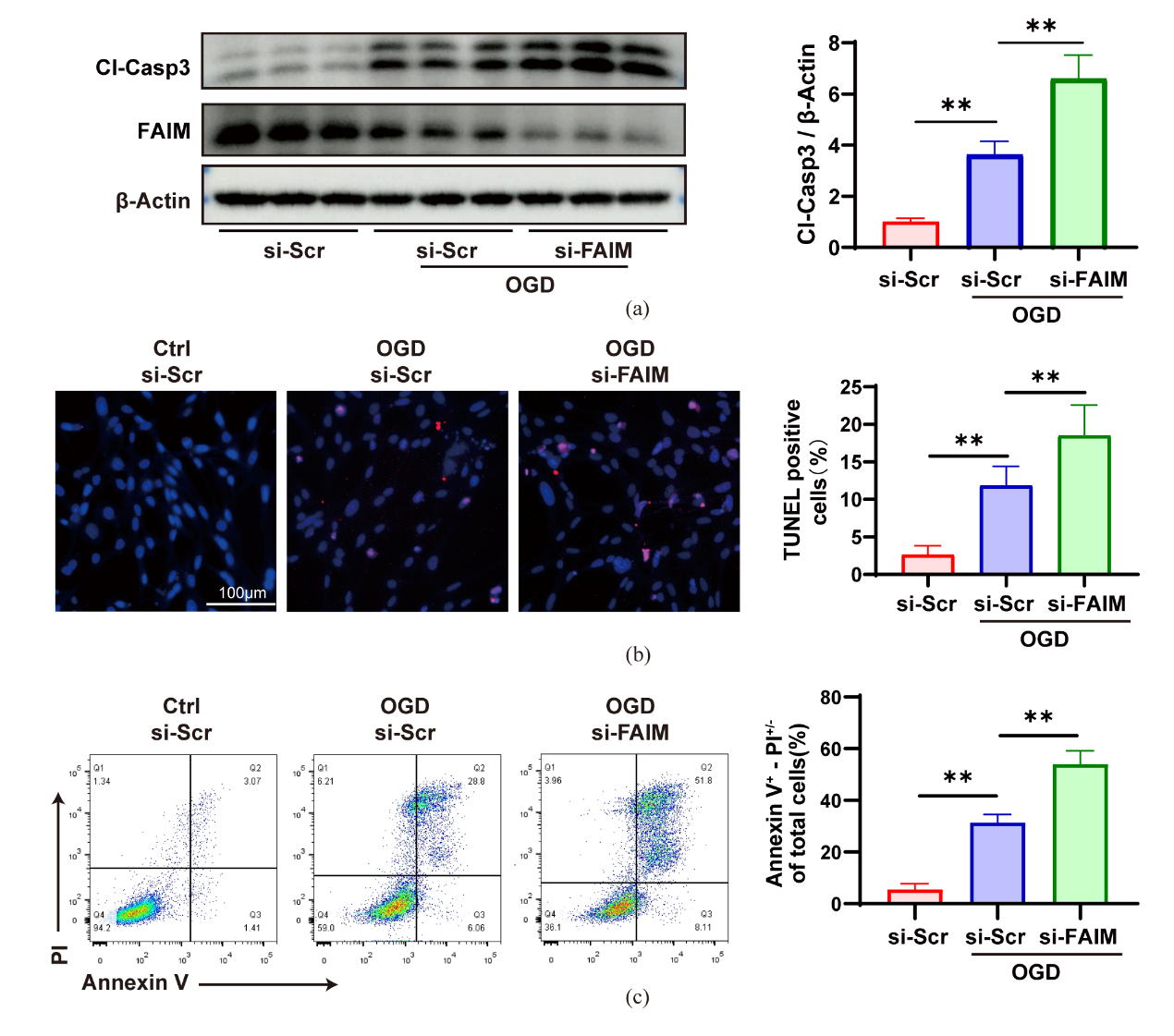


**Supplementary Figure S5 FAIM knockdown facilitated MSCs apoptosis**

(a) Cleaved caspase-3 protein levels after siRNA-mediated FAIM knockdown or scramble control administration followed by OGD treatment. The results of densitometric quantitation are shown on the right. (b) TUNEL staining after siRNA-mediated FAIM knockdown or scramble control administration followed by OGD treatment for 12 h (scale bar=100 μm). Quantitative results are shown on the right. Six visual fields were randomly chosen for each well; the apoptotic index was determined as the percentage of TUNEL-positive nuclei. (c) Annexin V-APC/PI staining was performed to determine the apoptosis rate. The data are shown as the mean ± SD, ** denotes P < 0.01.


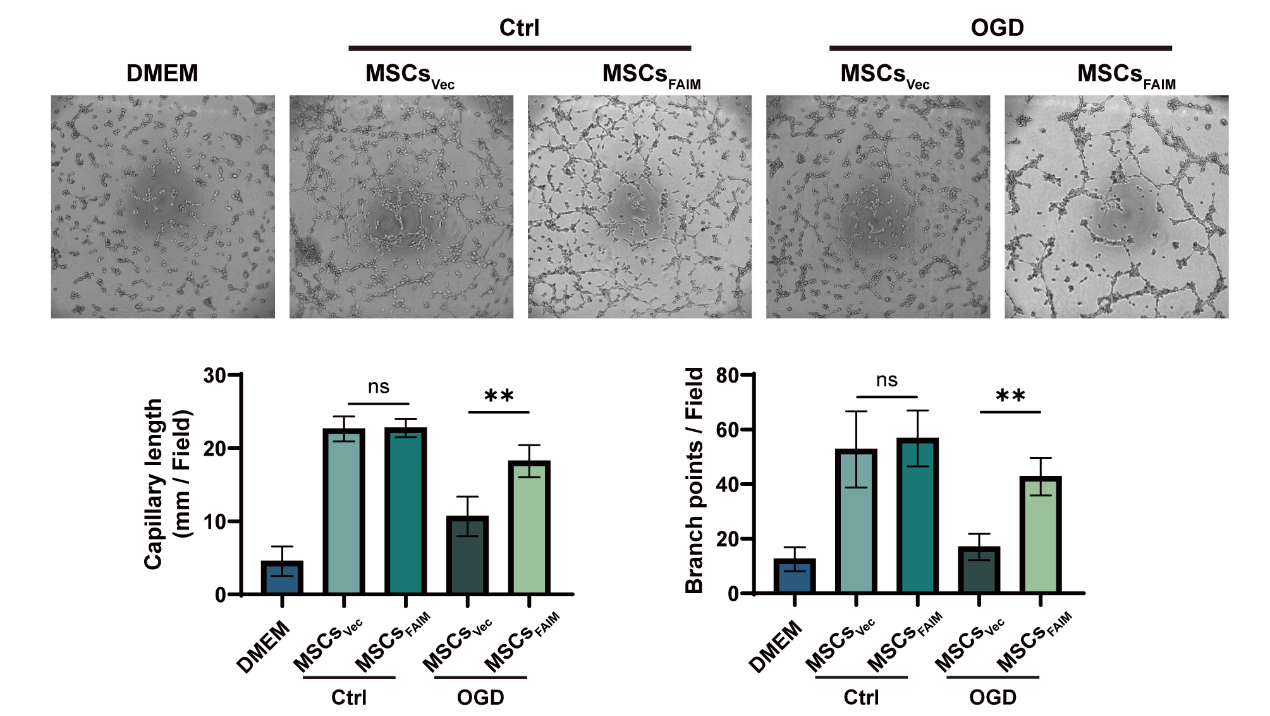


**Supplementary Figure S6 FAIM overexpression reversed the decrease in the proangiogenic capacity of conditioned medium from MSCs induced by OGD**

MSCs_Vec_ and MSCs_FAIM_ were exposed to OGD conditions or normoxia for 12 h, and conditioned medium was obtained and used to culture HUVECs in Matrigel-coated 96-well plates. The conditioned medium was normalized to contain 1 × 10^6 MSCs, and tube formation was observed 6 h after seeding. Capillary length and branch points were quantified with ImageJ software using the angiogenesis analyzer plugin (n = 4). The data are shown as the mean ± SD. ns indicates not significant, ** P < 0.01.
